# Self-Care Agency in Cardiovascular Care: A Cross-Sectional Study on the Interplay Between Self-Efficacy, Loneliness and Physical Activity

**DOI:** 10.3390/healthcare14050581

**Published:** 2026-02-25

**Authors:** Tino Prell, Lisa Bauer, Roland Prondzinsky, Aline Schönenberg

**Affiliations:** 1 Department of Geriatrics, Jena University Hospital, 07747 Jena, Germany; 2 Department of Geriatrics, Halle University Hospital, 06120 Halle (Saale), Germany; 3 Clinic for Psychiatry, Psychotherapy and Psychosomatics, Section Psychocardiology, Carl-von-Basedow Hospital, 06217 Merseburg, Germany

**Keywords:** loneliness, self-care, cardiovascular disease, CVD, self-efficacy, older adults

## Abstract

Background: Appraisal for self-care agency (ASAS) is central to cardiovascular disease (CVD) management, yet the influence of loneliness as a social stressor remains under-characterized. Methods: In patients with CVD (*N* = 80), cross-sectional predictors for ASAS were assessed via ordinary least squares (OLS) and quantile regression; robust methods are supplemented to buffer the sample size. Interaction effects on ASAS were tested between loneliness and physical activity, and loneliness and self-efficacy. Results: The diminishing effect of loneliness explained 10% of ASAS variance and remained significant when controlling for covariates (ß = −1.05, *p* = 0.031). Self-efficacy (β = 2.97, *p* = 0.009) and physical activity (β = 5.13, *p* = 0.001) were positively associated with ASAS, although quantile models indicated heterogeneity: loneliness exerts its effect at low ASAS (τ = 0.25; ß = −1.95, *p* < 0.001) whereas physical activity is influential at high ASAS (τ = 0.75; ß = 5.21, *p* < 0.001) and self-efficacy across all levels. Interactions showed a buffering of loneliness’ negative influence on ASAS via physical activity (ß = 3.29, *p* = 0.044). Conclusions: Self-efficacy is crucial for self-care agency in CVD. While loneliness exerts its strongest detriment at lower self-care levels, physical activity may attenuate loneliness-related vulnerability. These findings support interventions enhancing self-efficacy, promoting activity, and targeting loneliness to improve self-care.

## 1. Introduction

Effective self-care is a cornerstone of managing chronic illnesses, particularly cardiovascular diseases (CVD). According to the World Health Organization (WHO), self-care in health describes the general ability to promote and maintain a certain health status, prevent illness, or cope appropriately [1]. Actions for self-care are numerous and vary from person to person but generally include lifestyle- and health-related habits of all kinds. For example, physical activity is an important self-care action reducing mortality risk for various illnesses, while the monitoring of blood pressure is crucial for hypertension patients [1]. Especially for individuals with cardiovascular conditions, self-care is a crucial element in the prevention of complications, the management of symptoms, and the maintenance of quality of life [2,3]. The capacity for self-care (self-care agency, ASAS) has been linked to better health outcomes in chronic disease populations [4,5]. In heart failure, for example, patients who practice adequate self-care experience fewer symptoms and improved well-being compared to those with poor self-care [6]. An expanding body of research has highlighted the significant advantages of self-care interventions ranging from patient education to lifestyle coaching [7,8,9], which has ultimately led to the release of the WHO Guideline on Self-Care Interventions [10,11]. Encouraging self-care not only benefits individual patients but also addresses broader public health goals by improving quality of life and easing the burden on healthcare systems. A critical factor that influences self-care agency is self-efficacy. The concept of self-efficacy, introduced by Bandura (1977), refers to an individual’s belief in their own ability to successfully execute the behaviours necessary to achieve specific outcomes [12]. In essence, it signifies a sense of assurance in one’s ability to exert control over one’s own motivations, behaviour, and environment [13]. Self-efficacy represents a fundamental psychological construct that impacts an individual’s cognitive processes, emotional state, and behavioural responses, particularly in the context of challenging circumstances. A high level of self-efficacy is therefore associated with greater adherence to self-care practices, as individuals with greater confidence in their abilities are more likely to take proactive measures to manage their health [14,15,16]. In the context of cardiovascular disease, self-efficacy can be a pivotal factor in effective disease management, influencing behaviours such as medication adherence, dietary modifications, physical activity, and regular monitoring of symptoms [17]. Thus, self-efficacy is a cornerstone in promoting self-care that can be targeted in clinical interventions, as previous interventions that bolster self-efficacy have shown success in improving patients’ self-care and clinical outcomes [18]. The relationship between self-efficacy and self-care agency is therefore of particular interest in understanding how to promote better health outcomes in this population [17]. In the context of cardiovascular disease, self-efficacy can dictate the transition from passively suffering symptoms to actively adjusting lifestyle and seeking timely care [18].

Self-efficacy can be influenced by various factors. It is essential to know these factors, as these need to be addressed in interventions to improve self-care. Demographic parameters such as age and gender may exert a significant influence on the development of self-care behaviours and capabilities [3,17,19,20]. However, less is known about the influence of loneliness on self-care and self-efficacy in chronically ill patients. First results are contradictive and indicate effects specific to patient groups, with Masoudi et al. [21] showing a strong negative association between loneliness and self-care in community-dwelling older adults, whereas Nejadsadeghi et al. [22] showed a positive link between the variables in patients with chronic kidney disease. Generally, loneliness is often linked to worse physical health outcomes, depression and cognition, but less so to other psychological variables [23]. As part of the broader construct of social connectedness [24], loneliness is defined as the distressing subjective feeling of insufficient companionship [25]. This psychosocial stressor is common in chronic illness; a recent review reported that between 5% and 65% of heart disease patients experience loneliness [25]. As loneliness can be considered an emotional and physical stressor, accumulating evidence links loneliness to adverse health outcomes, especially regarding CVD [26,27]. Individuals who feel lonely are at elevated risk for incident CVD and worse prognoses [28,29]; even among patients with established cardiovascular conditions, those reporting higher loneliness have higher rates of cardiac events and mortality, with a recent review citing a hazard ratio of up to 1.46 [30]. Notably, these associations persist, even after adjusting for depression and biomedical risk factors [31]. In a longitudinal study on women, Chang and colleagues [32] even report elevated risk for fatal and non-fatal myocardial infarction for socially isolated women in comparison to well-integrated women. Such findings underscore that loneliness is a potent psychosocial determinant of cardiovascular health in its own right [28]. Lonely patients often lack the emotional encouragement or practical help that comes from social support networks, potentially making it harder to follow treatment plans [33]. Indeed, robust social support has been shown to bolster self-care confidence and maintenance in heart failure [34]. Loneliness is also closely intertwined with psychological functioning, which in turn can impact self-care. Lonely individuals are more prone to depression, anxiety, and lower psychological well-being [35]. In patients with CVD, this psychosocial burden may erode the motivation and cognitive resources needed for effective self-care [34]. Conversely, the absence of supportive relationships—as reflected by loneliness—can plausibly undermine patients’ capacity to perform beneficial health behaviours [36]. Taken together, these observations suggest that loneliness may negatively affect self-care through increased psychological distress and reduced external support, thereby impacting clinical outcomes.

Given this background, the present study includes loneliness and self-efficacy as variables influencing self-care agency in CVD patients. Patients who feel lonely are expected to exhibit a lower capacity to manage their health, after accounting for other factors. By integrating loneliness into our predictive model, we aim to shed light on this often-neglected psychosocial determinant of self-care. Establishing a link between loneliness and self-care agency would not only deepen our understanding of the challenges faced by CVD patients but also underscore the need to address social well-being in interventions to improve chronic illness self-management. Likewise, we expect patients with low self-efficacy to struggle with self-care, opening up an intervention path promoting psychological support to foster faith in one’s abilities. Such insights align with a broader emphasis on holistic care, recognizing that improving patients’ social and emotional support could enhance their self-care behaviours and ultimately cardiovascular outcomes [37]. We anticipate that our findings will contribute to the growing literature on how psychosocial determinants like loneliness impact chronic illness self-management and may inform future interventions aimed at enhancing self-care by addressing patients’ social connectedness.

## 2. Materials and Methods

### 2.1. Study Design and Sample

This cross-sectional observational study was conducted at the Department of Cardiology at the Carl-von-Basedow Hospital in Merseburg, Germany. Data collection took place in January 2025. Written informed consent was obtained from all patients. The study was approved by the local ethics committee of the University Hospital, given on 30 October 2024 (2024-164). Patients were randomly selected from the department wards. Of 102 initially included participants, 80 completed the self-care questionnaire as a key measure of interest and were thus included in the analysis. A post hoc sample size calculation is given in the Supplementary Materials in Supplement Section S1, indicating that effects with medium effect size around 0.20 can be detected with our data, and a STROBE checklist is given in Section 4.

### 2.2. Dependent Variable: Self-Care Agency

The German version of the Appraisal for Self-Care Agency Scale (ASAS-R) was the main measurement of interest [38]. It comprises 15 items posed on a 5-point Likert scale ranging from 1 = totally disagree to 5 = totally agree. Items 4, 11, 14, and 15 of the ASAS-R were reverse-coded and thus re-scaled to match the scale of the other items. Total scores range from 15 to 75, and higher scores indicate greater self-care agency.

### 2.3. Independent Variables

- Sex (1 = female, 2 = male)
- Age (years)
- Self-efficacy: The Short Scale for Measuring General Self-Efficacy Beliefs (ASKU) is a brief, three-item tool developed to assess an individual’s belief in their ability to effectively handle challenges and achieve goals. It is reliable, shows strong construct validity, and is well-suited for use in large-scale surveys and interdisciplinary research due to its concise format. Despite its brevity, it captures general self-efficacy accurately and performs consistently across different survey methods. The survey utilized a five-point Likert scale (from 1 = ‘strongly disagree’ to 5 = ‘strongly agree’). To derive a composite score, the responses to all individual items were averaged. The resulting scale value ranges from 1 to 5, with higher values indicating higher self-efficacy [39].
- Psychological distress (SCL) measured with the Mini-Symptom-Checklist (Mini-SCL): It enquires after 18 common physical and psychological symptoms on a five-point scale, rating how much patients have suffered from each symptom over the last seven days [40].
- Loneliness as measured with the 3-Item UCLA Loneliness Scale rated on a four-point Likert scale, culminating in a sum-score between 3 and 12 points. Higher scores indicate higher levels of loneliness [41].

To describe the cohort in more detail, we also collected the following variables:

- education level according to the German education system: low (no degree or up to 8 years), middle (10 years), high (13 years corresponding to A-Levels, university degree)
- employment status: employed (part-time or full-time), unemployed, retired
- living situation: living alone, living with somebody. Previous research indicates that perceived loneliness and living alone are distinct; their co-occurrence may amplify deficits in practical and emotional support, thus living alone is hypothesized to strengthen the association between loneliness and self-care agency [42]
- regular physical activity of at least 30 min/day (yes/no). Physical activity can buffer psychosocial stressors via affect regulation and social connectedness; as many physical activities are performed in groups or pairs and physical activity is known to improve sleep and mood [43], we expect being active to attenuate the adverse association between loneliness and self-care agency [44]
- Health Satisfaction (HS) as measured by item 2 of the WHOQOL-Bref questionnaire. This item enquires after the subjective satisfaction with the current state of health on a five-point Likert scale ranging from 1 = very dissatisfied to 5 = very satisfied [45].

In summary, the present manuscript treats ASAS as a key outcome for cardiovascular patients. All analyses are controlled for standard sociodemographic information such as age, gender, education and living conditions. Additionally, we propose the following variables as predictors for ASAS:

- ASKU, as self-efficacy can be considered a necessary pre-requisite for self-care
- SCL, because mood and symptom burden may influence an individual’s capacity for self-care; for example, higher depressiveness may attenuate self-care agency due to motivational deficits
- Loneliness, as social context is practically linked to self-care in the sense of having physical help, but it also reflects a deeply personal feeling linked with self-worth and mood
- Health satisfaction, as satisfaction with one’s own health may determine the amount of self-care a person deems necessary for themselves
- Physical activity as a reflection of fitness and physical care; we additionally expect physical activity to buffer the negative effects of the other above-mentioned psychosocial and health-related predictor variables and thus include physical activity both as a predictor variable and a moderator variable.

### 2.4. Statistical Analyses

All analyses were conducted in R Version 4.5.1 (R Foundation for Statistical Computing [46]); all tests were two-sided, with statistical significance indicated at *p* < 0.05. We initially provide an overview of the included participants with descriptive statistics, in which continuous variables were summarized as mean (SD) and categorical variables as n (%).

To select predictors of self-care agency (ASAS) while balancing parsimony and overfitting with mixed predictor types, we first fit an elastic-net regularization with α = 0.50 and 10-fold cross-validation using the R-package glmnet [47]. Elastic net selects variables based on their reduction in the prediction error. Factors were dummy coded and the cross-validated λmin defined the non-zero coefficient set. These predictors were then re-estimated in a conventional ordinary least squares (OLS) regression model to obtain interpretable coefficients and global fit indices. To examine distributional robustness and effect heterogeneity across ASAS, we estimated quantile regressions at τ = 0.50 (median), τ = 0.75 (upper quartile) and τ = 0.25 (lower quartile) with the same predictor set defined in the elastic net regularization (package quantreg [48]).

We tested for heteroscedasticity with the Breusch–Pagan test and multicollinearity was assessed via VIF/GVIF. Heteroscedasticity-robust inference test and cubic-spline specifications were performed [49] to ensure robustness of the results (Supplement Section S2).

To probe effect modification and plausible departures from linearity in predictors of ASAS, we estimated a moderation model and a spline sensitivity model [50]. For ease of interpretation, in the moderation analysis. continuous psychosocial predictors were z-standardized to yield coefficients per 1 SD and to reduce collinearity in interaction terms. The variables identified in the elastic net regularization were entered into the model with main effects, with additional interaction terms to reflect previous results and indicate the inter-relation between the relevant variables: zUCLA*physical activity, and zUCLA*zASKU. To examine potential nonlinearity in key continuous predictors, we fitted natural cubic splines (df = 3) for numeric variables within an otherwise analogous linear model and compared this to the linear specification using a likelihood-ratio (extra-df) F-test and Akaike’s Information Criterion (AIC). Given the modest sample and to avoid overfitting, we limited flexibility to df = 3 per predictor and treated any incremental fit as supportive only if both the likelihood-ratio test and information criteria indicated a clear advantage for the spline specification (Supplement Section S3).

All Models were estimated on complete cases for the variables involved (listwise deletion).

## 3. Results

The sample was approximately sex-balanced, predominantly retired, and physically activity was uncommon. Less than a third of patients lived alone, and educational attainment spanned low, medium, and high categories (Table 1). ASAS values were broadly distributed within the possible range; UCLA was concentrated near its lower bound (floor tendency, Table 1, Supplement Figure S1).

### 3.1. Predictors of ASAS

As a first step, we performed a simple linear regression containing only the UCLA sum score as independent variable to predict the ASAS in order to determine the strength of their association. In the model, the UCLA alone explained 10% of ASAS variance (β = −1.49, 95% CI [−2.45, −0.53], SE = 0.48, *p* = 0.003, R^2^ = 0.11, adj R^2^ = 0.10). This significant inverse relation between UCLA and ASAS remained when adding sociodemographic variables to the model (β = −1.55, 95% CI [−2.45, −0.55], SE = 0.48, *p* = 0.003, adj R^2^ = 0.13).

Lastly, to determine the robustness of the UCLA’s influence after including the additional psychosocial variables, we performed an OLS model following variable selection via elastic net regularization with 10-fold cross validation. In the elastic-net model (λmin = 0.894, R^2^ of approximately 0.31) the non-zero coefficients selected age, UCLA, ASKU, living alone, education (high), job (employed), and physical activity (yes). Refitting these terms in an OLS model produced a statistically significant specification, F(10, 69) = 3.45, R^2^ = 0.333 (adjusted 0.237; AIC = 537.2). VIF for all included variables was ≤2.6 indicating no collinearity, and Breusch–Pagan test confirmed homoscedasticity (BP = 10.9, *p* = 0.29). In this mean-based refit, the UCLA remained inversely associated with ASAS (β = −1.05, 95% CI [−2.00, −0.10], SE = 0.48, *p* = 0.031), whereas ASKU (β = 2.97, 95% CI [0.76, 5.17], SE = 1.10, *p* = 0.009) and physical activity (β = 5.13, 95% CI [0.65, 9.62], SE = 2.25, *p* = 0.026) were positively associated with ASAS. Age, SCL and the remaining sociodemographic variables were not statistically significant (Table 2).

Because UCLA was skewed and the modelling sample was modest (n = 80), we complemented the mean-based analysis with robust distribution-aware checks. The spline model did not improve fit (Δdf = 4, F = 1.85, *p* = 0.131; AIC 536.6 vs. 537.2) and the associations with the included variables did not change, see Supplement Section S2.

**Figure 1:**
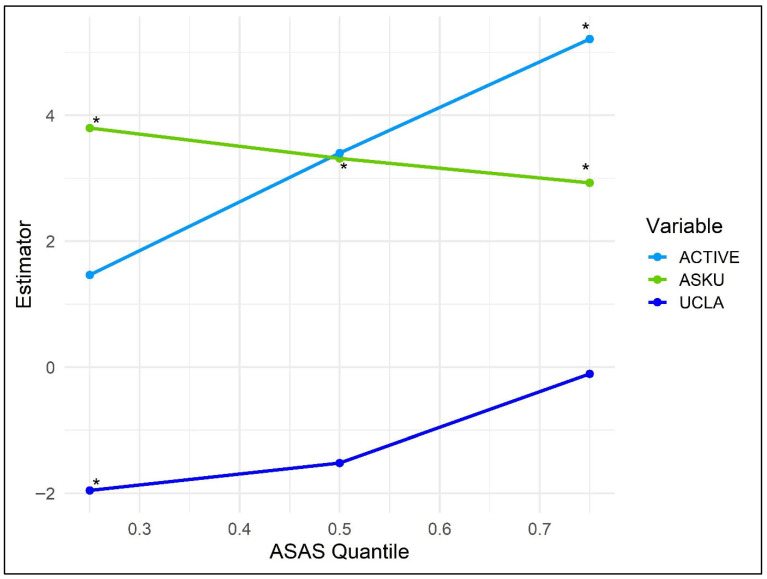
Quantile regression on ASAS using τ = 0.25, τ = 0.50 and τ = 0.75 based on Table 3 Note: ACTIVE = physical activity, ASKU = self-efficacy, UCLA = loneliness. * indicates significance *p* < 0.05.

Next, quantile regression was used to examine effect heterogeneity across the ASAS distribution. For this purpose, the selected variables from the elastic net regularization were entered into the model (Table 3, Figure 1). At the lowest quartile of ASAS (τ = 0.25, pseudo-R^2^ = 0.42), UCLA (ß = −1.95, 95% CI [−2.90, −1.00], *p* < 0.001) and ASKU (ß = 3.80, 95% CI [0.92, 6.68], *p* = 0.010) were significantly associated with ASAS, whereas physical activity was not (*p* = 0.748). At the median of the ASAS (τ = 0.50, pseudo-R^2^ = 0.16), only the ASKU retained its significant association (ß = 3.31, 95% CI [0.45, 6.18], *p* = 0.024). In contrast, at the upper quartile (τ = 0.75, pseudo-R^2^ = 0.27), physical activity obtained significance (ß = 5.21, 95% CI [2.30, 8.12], *p* = 0.001) while the ASKU retained its influence (ß = 2.93, 95% CI [1.15, 4.71], *p* = 0.002) and UCLA remained insignificant (*p* = 0.903). These findings indicate that loneliness is particularly influential among participants with low self-care agency, whereas physical activity exerts its clearest benefit at the upper end of the ASAS distribution and ASKU is continually influential.

### 3.2. Moderation

As a last step, we aimed to detect potential moderation effects between UCLA and ASAS depending on the other variables in the model. To assess effect modification and potential departures from linearity in predictors of ASAS, we fit a moderation model and a spline sensitivity model based on the above-specified results. In the moderation model, the significant z-standardized psychosocial predictors (zUCLA, zASKU) in addition to physical activity and the sociodemographic variables from elastic net regularization were entered with main effects on ASAS. Interaction effects were specified between zUCLA*physical activity and zUCLA*zASKU.

**Figure 2:**
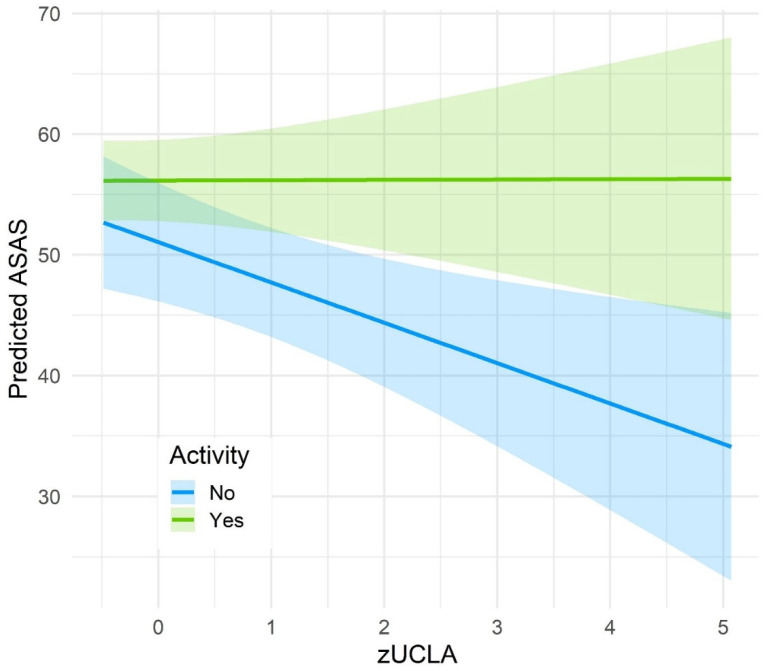
Interaction effect between loneliness (UCLA) and self-care agency (ASAS) depending on physical activity.

The model (adj. R^2^ = 0.28) retained the previously identified direct effects of UCLA, ASKU and physical activity on ASAS (Table 4). Even when considering interaction effects, the significant main effect of zUCLA (est = −3.36, 95% CI [−5.72, −0.99], *p* = 0.006) indicates that higher loneliness is associated with lower self-care, whereas increased activity (est = 4.94, 95% CI [0.56, 9.32], *p* = 0.028) and higher self-efficacy (est = 2.57, 95% CI [1.05, 4.10], *p* = 0.001) are linked with better self-care. This means that with each 1 SD increase in UCLA, ASAS is reduced by 3.36 points, whereas each SD increase in self-efficacy increases ASAS by 2.57 points. Of note, while there was no interaction between UCLA and ASKU on ASAS, the interaction between UCLA and physical activity was statistically significant (est = 3.29, 95% CI [0.08, 6.49], *p* = 0.044). These results indicate that the negative effect of UCLA on ASAS is buffered by physical activity (Figure 2).

A spline sensitivity analysis (Supplement Section S3) did not improve model fit.

## 4. Discussion

The aim of the present study was to understand the capacity for self-care in patients with CVD, as self-care is a crucial cornerstone of symptom management and quality of life maintenance [2,3]. Psychosocial stressors are now increasingly recognized in their influence on cardiovascular health [51], and loneliness can be considered a strong psychosocial stressor influencing CVD due to its associations with inflammatory processes [26,27].

In three steps, we aimed to (1) assess the influence of loneliness on self-care agency (ASAS) in patients with CVD while controlling for other psychosocial and sociodemographic variables, (2) understand in-depth whether these associations differ at varying levels of ASAS, and (3) assess potential moderating effects between loneliness and other variables on ASAS.

First, in a simple regression model, loneliness alone explained around 10% of ASAS variance, constituting a small-to-medium effect size. In comparison, after adding sociodemographic and psychosocial variables, the explained variance rose up to 25%, indicating that loneliness explains a substantial portion of ASAS variance in comparison to the six additional variables that were added in the full model. This association between loneliness and ASAS remained even after elastic net regularization and in the presence of other psychosocial and sociodemographic variables. In previous studies, this association between self-care and loneliness has been confirmed: in cross-sectional data on older adults, Masoudi, Sarbazi, Soleimanpour, Abbasian, Ghasemi, Rostami, Azizi and Soleimanpour [21] found a negative association between self-care and loneliness, and in a recent review, Puyané et al. [52] conclude that loneliness is linked to poorer self-care abilities.

As self-care is a crucial cornerstone in the management of both illness and stress, its reduction by loneliness may help explain the strong influence of loneliness on CVD [28,29,30]: as indicated by previous research, loneliness operates both as a psychosocial stressor on a biological level [32], and as a potential inhibitor of self-care in CVD patients as described in our data. However, the exact mechanisms between loneliness and self-care must be causally determined in longitudinal data.

A lack of both practical help and encouragement from a social system in lonely persons may partially explain this effect [33], and potential mental health problems arising due to loneliness [35] may further reduce motivation and ability to perform self-care [34]. Taken together, these results imply that the negative effect of loneliness on self-care may arise through biological and behavioural mechanisms, especially mental health problems and reduced support [51].

Notably, the effect of loneliness on self-care ability remained significant irrespective of self-efficacy and physical activity [31], which further independently influenced self-care. As depressiveness was not retained in the model after elastic net, it can be assumed that in our data, it did not contribute to self-care ability above and beyond the included variables; however, this effect should be confirmed in larger datasets.

Depressiveness is known as a common factor influencing both loneliness and self-care [35,53], as described above. Our results suggest that loneliness is negatively associated with self-care irrespective of co-occurring depressiveness. However, due to the cross-sectional nature of our data, no conclusion on causality can be drawn and longitudinal data is needed to confirm the results.

In contrast, self-efficacy had a profound positive association with self-care in our data. Self-efficacy is often cited as a cornerstone of self-care and self-management abilities: in a review on hypertensive patients, Tan et al. [54] conclude that higher self-efficacy is linked with better self-care and self-management ability, and previous concept analyses on self-care and self-management describe self-efficacy as indispensable for self-care [37,55]. As self-efficacy describes the belief in one’s abilities to handle a situation and achieve the desired outcome, being convinced that the own behaviour can improve health is crucial for self-care [12,13,14]. This importance of self-efficacy as a foundation for self-care is mirrored in the results obtained in our quantile regression, showing the association between higher self-efficacy and higher self-care for all levels of ASAS.

In contrast, the quantile regression suggests that in our data, the negative effect of loneliness on self-care agency is primarily driven by a strong association at low ASAS levels: those who have low self-care agency appear particularly vulnerable to the effect of loneliness, while at higher levels of self-care, loneliness appears to be compensated. Notably, as a limitation of the present data with its cross-sectional design and moderate sample size, alternative explanations such as floor effects or regression to the mean must be considered, and longitudinal data in larger samples is needed to understand the nuances of self-care agency and its association with psychosocial and health-related factors.

The results from our moderation analysis may shed a light on this association by highlighting an interaction between loneliness and physical activity: engaging in physical activity may buffer the negative effect of loneliness on self-care agency. As physical activity exerted its strongest influence at high ASAS levels, it appears possible that for persons with already low self-care agency, loneliness further diminishes self-care. In contrast, physical activity may attenuate the effect of loneliness on self-care at higher self-care levels. However, due to the cross-sectional data, no direction of effects can be determined, and it may also be the case that persons at high self-care agency generally perform more physical activity and are less lonely due to participating in sports groups. Likewise, the interaction effect is only marginally significant (*p* = 0.044) in a relatively small sample size. In future studies, the effect of activity on the association between loneliness and self-care must be determined longitudinally.

This effect of physical activity on self-care is well-documented. While physical activity itself may be considered an active form of self-care, it is also known to boost self-esteem and motivation for further self-care activities while reducing stress perception [56,57]. Additionally, this inverse association between loneliness and physical activity may also be rooted in practical reasons: loneliness may be associated with a reduced social circle, and many physical activities such as walking and sports are typically enjoyed with others; thus, the capacity for beneficial health behaviours may be reduced in lonely persons [36,58]. In a longitudinal study on coronary heart disease in women [32], the authors conclude that the increased risk for myocardial infarction is delivered both via biological and behavioural mechanism related to beneficial health behaviour.

Taken together, our results highlight loneliness as a serious inhibitor of self-care agency in patients with CVD, especially for those already vulnerable patients at low self-care levels. In addition to strengthening self-efficacy and fostering motivation for self-care, our results suggest that promoting physical activity may attenuate the effect of loneliness on self-care and thus improve not only self-care itself but also health outcomes. While these psychosocial parameters are already increasingly incorporated in the care for patients with CVD, their influence cannot be emphasized enough; in addition to traditional medical care, psychosocial pathways to health via motivation and health behaviour must be considered, and patients must be supported in their ability to care for themselves to achieve best health outcomes.

### Limitations

While this study sheds light on first results regarding the association between loneliness as an important psychosocial stressor and self-care as a cornerstone of health management in cardiovascular disease, it is not free of limitations.

First, the cross-sectional data precludes any interpretation of causality. Longitudinal data is required to uncover the direction of effects between loneliness, self-care and the associated variables, and to confirm the robustness of the results. Likewise, the sample size was small, although robust methodological approaches were supplemented to arrive at the most reliable results despite the limited patient number. In concordance, the monocentric study design and the selective study cohort of older patients with cardiovascular illness inhibits the generalizability of the results. Additionally, the UCLA showed a floor tendency with limited variance in our study cohort, potentially attenuating the effects. In future studies, the analyses should be repeated in a cohort with sufficient variance in loneliness scores to confirm the relationships. Likewise, as behavioural data is oftentimes interconnected and the included variables cannot be statistically independent of one another, the moderation analysis should be interpreted in the light of an interconnectedness of all variables.

Lastly, the study relied primarily on self-report measures regarding the key variables. Although loneliness, depressiveness, self-efficacy and self-care are by their definition subjective experiences and can only be measured subjectively, self-report instruments may be prone to bias. Especially the reliance on the same measurement approach for outcome and predictor variables may result in bias due to common method variance. Therefore, future studies should repeat the present analyses with different measurement instruments to confirm the soundness of the results.

## 5. Conclusions

In patients with cardiovascular disease, loneliness as a psychosocial stressor reduces self-care agency. Especially for persons at the lower spectrum of self-care agency, additional loneliness further reduces self-care abilities. Notably, at higher self-care levels, this diminishing effect of loneliness on self-care agency is buffered by physical activity. These results suggest that loneliness must be taken into consideration in the treatment of cardiovascular illnesses, and longitudinal studies should be designed to fully understand its association with self-care and cardiovascular diseases to arrive at effective intervention strategies. Based on this preliminary cross-sectional data, the upkeep of self-efficacy as well as partaking in physical activity appear as beneficial pathways to improve self-care agency in cardiovascular disease.

## Figures and Tables

**Table 1:** Characteristics of patients (N = 80).

Variable	Mean (SD)	Range
Age	70.6 (10.7)	37–88
ASAS	54.3 (7.4)	28–70
ASKU	4.2 (0.7)	2–5
UCLA	3.8 (1.6)	3–12
SCL	9.4 (7.7)	0–33
	**Count**	**Percentage**
Sex: Female	41	51.25
Sex: Male	39	48.75
Living: Not Alone	57	71.3
Living: Alone	23	28.7
Education: low	21	26.2
Education: medium	39	48.8
Education: high	20	25.0
Employment: retired	64	80.0
Employment: employed	11	13.8
Employment: unemployed	5	6.2
Physical Activity: yes	11	13.8
Physical Activity: no	69	86.2
HS: very dissatisfied	6	7.5
HS: dissatisfied	38	47.5
HS: neither	8	10.0
HS: satisfied	18	22.5
HS: very satisfied	10	12.5

ASAS = Appraisal for self-care agency scale, ASKU = Short scale for measuring general self-efficacy beliefs, UCLA = UCLA loneliness scale, SCL = Mini-symptom-checklist, HS = Health satisfaction (WHOQOL-Bref Item 2).

**Table 2:** OLS regression model after elastic net regularization.

	ASAS		
Predictors	Estimates	CI	*p*
(Intercept)	41.82	23.99–59.64	<0.001
Age	0.00	−0.22–0.22	0.994
UCLA	−1.05	−2.00–−0.10	0.031
ASKU	2.97	0.76–5.17	0.009
Living [Alone]	−1.35	−4.81–2.11	0.440
Education [Middle]	−0.07	−3.99–3.84	0.971
Education [High]	2.40	−1.71–6.51	0.248
Unemployed	−4.16	−10.09–1.78	0.167
Retired	0.09	−6.91–7.09	0.979
Physical Activity: Yes	5.13	0.65–9.62	0.026
Observations	80		
R^2^/R^2^ adjusted	0.333/0.247		

ASAS = Appraisal for self-care agency scale, ASKU = Short scale for measuring general self-efficacy beliefs, UCLA = UCLA loneliness scale, SCL = Mini-symptom-checklist. Originally included in the model but not retained after elastic net regularization: sex, SCL (Mini-symptom-checklist), health satisfaction (WHOQOL-Bref item 2).

**Table 3:** Quantile regression for ASAS sum score.

	Tau = 0.25			Tau = 0.50			Tau = 0.75		
Variable	Est	CI	*p*	Est	CI	*p*	Est	CI	*p*
(Intercept)	52.82	30.14–75.49	<0.001	48.20	27.40–69.0	<0.001	32.32	15.55–49.09	<0.001
UCLA	−1.95	−2.90–−1.00	<0.001	−1.52	−3.31–0.27	0.095	−0.11	−1.82–1.60	0.903
ASKU	3.80	0.92–6.68	0.010	3.31	0.45–6.18	0.024	2.93	1.15–4.71	0.002
Activity	1.47	−7.60–10.53	0.748	3.40	−2.90–9.71	0.286	5.21	2.30–8.12	0.001
Age	−0.16	−0.44–0.13	0.282	−0.05	−0.26–0.16	0.645	0.13	−0.04–0.29	0.138
Living	−3.58	−8.67–1.51	0.166	−0.85	−5.96–4.26	0.742	0.91	−2.77–4.59	0.623
Education: middle	0.22	−5.88–6.32	0.943	−1.65	−14.3–1.64	0.402	0.11	−3.97–4.20	0.956
Education: high	3.07	−2.34–8.47	0.262	1.25	−3.17–5.68	0.574	3.36	−1.44–8.16	0.168
Unemployed	−11.0	−20.27–−1.79	0.020	−6.33	−14.44–1.78	0.124	2.05	−3.58–7.67	0.470
Retired	−1.02	−14.08–12.03	0.876	0.833	−4.42–6.90	0.757	−2.70	−13.04–7.64	0.604

Activity: Physical activity yes, ASAS = Appraisal for self-care agency scale, ASKU = Short scale for measuring general self-efficacy beliefs, UCLA = UCLA loneliness scale, SCL = Mini-symptom-checklist. Originally included in the model but not retained after elastic net regularization: sex, SCL(Mini-symptom-checklist), health satisfaction (WHOQOL-Bref item 2).

**Table 4:** Moderation model.

	ASAS		
Predictors	Estimates	CI	*p*
(Intercept)	57.91	39.50–76.32	<0.001
zUCLA	−3.36	−5.72–−0.99	0.006
Activity	4.94	0.56–9.32	0.028
zASKU	2.57	1.05–4.10	0.001
Education: middle	−1.79	−5.87–2.30	0.386
Education: high	2.07	−1.95–6.10	0.308
Unemployed	−5.96	−11.89–−0.03	0.049
Retired	−1.14	−7.91–5.63	0.738
Age	−0.09	−0.32–0.14	0.444
zUCLA × Activity	3.29	0.08–6.49	0.044
zUCLA × zASKU	1.00	−0.55–2.55	0.203
Observations	80		
R^2^/R^2^ adjusted	0.372/0.281		

Activity: Physical activity yes, ASAS = Appraisal for self-care agency scale, ASKU = Short scale for measuring general self-efficacy beliefs, UCLA = UCLA loneliness scale, SCL = Mini-symptom-checklist.

## Data Availability

Due to the sensitive nature of the data collected directly from patients, data are available from the corresponding author upon reasonable request.

## Supplementary Materials

The following supporting information can be downloaded at: https://www.mdpi.com/article/10.3390/healthcare14050581/s1, Supplement Section S1. Sample size calculation; Supplement Section S2. Information on heteroscedasticity-robust inference test and cubic-spline specification; Supplement Section S3. Spline-sensitive analyses with nonlinearity. Supplement Section S4. STROBE checklist. Supplement Figure S1. UCLA Histogram.

## Abbreviations

The following abbreviations are used in this manuscript:

ASAS	Self-Care Agency
ASKU	General Self-efficacy Beliefs
CI	Confidence Interval (95%)
CVD	Cardiovascular Disease
OLS	Ordinary Least Squares
SCL	Psychological Distress according to Mini-SCL Mini Symptom Checklist
UCLA	UCLA Loneliness Scale
WHO	World Health Organization
