# Supplementary material for: Self-Care Agency in Cardiovascular Care: A Cross-Sectional Study on the Interplay Between Self-Efficacy, Loneliness and Physical Activity
Source: Healthcare (Basel). 2026 Feb 25;14(5):581. doi: 10.3390/healthcare14050581 (PMC12984293; doi:10.3390/healthcare14050581)

Supplement Section S1. Sample size calculation

We conducted a post hoc power appraisal for the complete-case cohort (N = 80) and the final OLS model with 10 predictor degrees of freedom at a two-sided α = 0.05. Under standard multiple-regression assumptions, this sample provides approximately 80% power to detect an overall, medium-sized association (about 13% explained variance in ASAS). For individual 1-df predictors, the minimum detectable unique contribution is roughly 10% of variance (partial correlation ≈ 0.32), indicating that smaller effects are likely underpowered. These estimates pertain to the unpenalized OLS refit; elastic-net selection lacks a directly comparable power metric, and HC3-robust inference and quantile models typically yield slightly wider intervals, rendering our conclusions conservative.

A sensitivity analysis was conducted to determine the minimum detectable effect size given the sample size (N = 80), an alpha level of .05, and a desired power of .80. The analysis indicated that the study was sufficiently powered to detect medium-sized effects ($f^2 \approx 0.20$) for individual predictors, whereas smaller effects may have gone undetected.

Supplement Section S2. Information on heteroscedasticity-robust inference test and cubic-spline specification

Because UCLA was skewed and the modeling sample was modest (n = 80), we complemented the mean-based analysis with robust and distribution-aware checks.

Given bounded/skewed distributions for some predictors (notably UCLA), we prespecified heteroscedasticity-robust inference for the OLS refit using HC3 standard errors using the packages sandwich and lmtest (Zeileis, Köll et al. 2020). As a functional-form sensitivity analysis, we replaced linear terms for UCLA, SCL, and age with restricted cubic splines (df = 3; R-package splines (Wang and Yan 2021)) and compared the spline model to the linear specification using likelihood-ratio tests and AIC.

Although a Breusch–Pagan test did not indicate heteroscedasticity (BP = 11.61, df = 10, p = .312), we applied HC3-robust standard errors as a conservative safeguard. Under HC3, the association of ASKU with ASAS remained statistically robust and clinically meaningful (β = 2.92, 95% CI [0.44; 5.40], p = .024). The coefficient for physical activity attenuated to marginal significance (β = 5.08, 95% CI [−0.63; 10.80], p = .086), and the inverse association of UCLA no longer met conventional thresholds (β = −1.05, 95% CI [−2.53; 0.44], p = .173). To probe potential curvature in key continuous predictors, we compared the linear model with a restricted-cubic-spline specification (df = 3 for UCLA, SCL, and age). The spline model did not improve fit materially over the linear form (Δdf = 4, F = 1.85, p = .131; AIC 536.6 vs. 537.2), supporting the adequacy of linear terms in the primary analysis.

Wang, W. and J. Yan (2021). "Shape-Restricted Regression Splines with R Package splines2." Journal of Data Science **19**(3).
Zeileis, A., S. Köll and N. Graham (2020). "Various Versatile Variances: An Object-Oriented Implementation of Clustered Covariances in R." Journal of Statistical Software **95**(1): 1-36.

Supplement Section S3. Spline-sensitive analyses with nonlinearity

In spline sensitivity analyses, allowing nonlinearity for UCLA, SCL, and age yielded an adjusted R^2 = 0.283; σ = 6.00; n = 82 for this specification). Compared with the linear model on the same variables, there was no improvement due to the spline model (p = .46, linear

AIC = 547.3 vs spline AIC = 551.8). These results suggest that curvature does not materially alter inference, supporting the adequacy of linear terms for these predictors in the present sample.

Supplement Section S4. STROBE Checklist.

	Item No	Recommendation	Page
**Title and abstract**	1	(*a*) Indicate the study's design with a commonly used term in the title or the abstract	1
		(*b*) Provide in the abstract an informative and balanced summary of what was done and what was found	1
**Introduction**			
Background/rationale	2	Explain the scientific background and rationale for the investigation being reported	1-3
Objectives	3	State specific objectives, including any prespecified hypotheses	3
**Methods**			
Study design	4	Present key elements of study design early in the paper	3
Setting	5	Describe the setting, locations, and relevant dates, including periods of recruitment, exposure, follow-up, and data collection	3
Participants	6	(*a*) Give the eligibility criteria, and the sources and methods of selection of participants	3
Variables	7	Clearly define all outcomes, exposures, predictors, potential confounders, and effect modifiers. Give diagnostic criteria, if applicable	3-4
Data sources/ measurement	8*	For each variable of interest, give sources of data and details of methods of assessment (measurement). Describe comparability of assessment methods if there is more than one group	*3-4*
Bias	9	Describe any efforts to address potential sources of bias	4-5
Study size	10	Explain how the study size was arrived at	3
Quantitative variables	11	Explain how quantitative variables were handled in the analyses. If applicable, describe which groupings were chosen and why	4-5
Statistical methods	12	(*a*) Describe all statistical methods, including those used to control for confounding	4-5
		(*b*) Describe any methods used to examine subgroups and interactions	n.a.
		(*c*) Explain how missing data were addressed	5

		(*d*) If applicable, describe analytical methods taking account of sampling strategy	n.a.
		(*e*) Describe any sensitivity analyses	4-5
**Results**			
Participants	13*	(a) Report numbers of individuals at each stage of study—eg numbers potentially eligible, examined for eligibility, confirmed eligible, included in the study, completing follow-up, and analysed	3
		(b) Give reasons for non-participation at each stage	3
		(c) Consider use of a flow diagram	n.a.
Descriptive data	14*	(a) Give characteristics of study participants (eg demographic, clinical, social) and information on exposures and potential confounders	5
		(b) Indicate number of participants with missing data for each variable of interest	
Outcome data	15*	Report numbers of outcome events or summary measures	
Main results	16	(*a*) Give unadjusted estimates and, if applicable, confounder-adjusted estimates and their precision (eg, 95% confidence interval). Make clear which confounders were adjusted for and why they were included	5-10
		(*b*) Report category boundaries when continuous variables were categorized	5-10
		(*c*) If relevant, consider translating estimates of relative risk into absolute risk for a meaningful time period	
Other analyses	17	Report other analyses done—eg analyses of subgroups and interactions, and sensitivity analyses	5-10, Suppl.
**Discussion**			
Key results	18	Summarise key results with reference to study objectives	10
Limitations	19	Discuss limitations of the study, taking into account sources of potential bias or imprecision. Discuss both direction and magnitude of any potential bias	12
Interpretation	20	Give a cautious overall interpretation of results considering objectives, limitations, multiplicity of analyses, results from similar studies, and other relevant evidence	12-13
Generalisability	21	Discuss the generalisability (external validity) of the study results	12-13
**Other information**			
Funding	22	Give the source of funding and the role of the funders for the present study and, if applicable, for the original study on which the present article is based	13

Supplement Figure S1. Histogram for UCLA

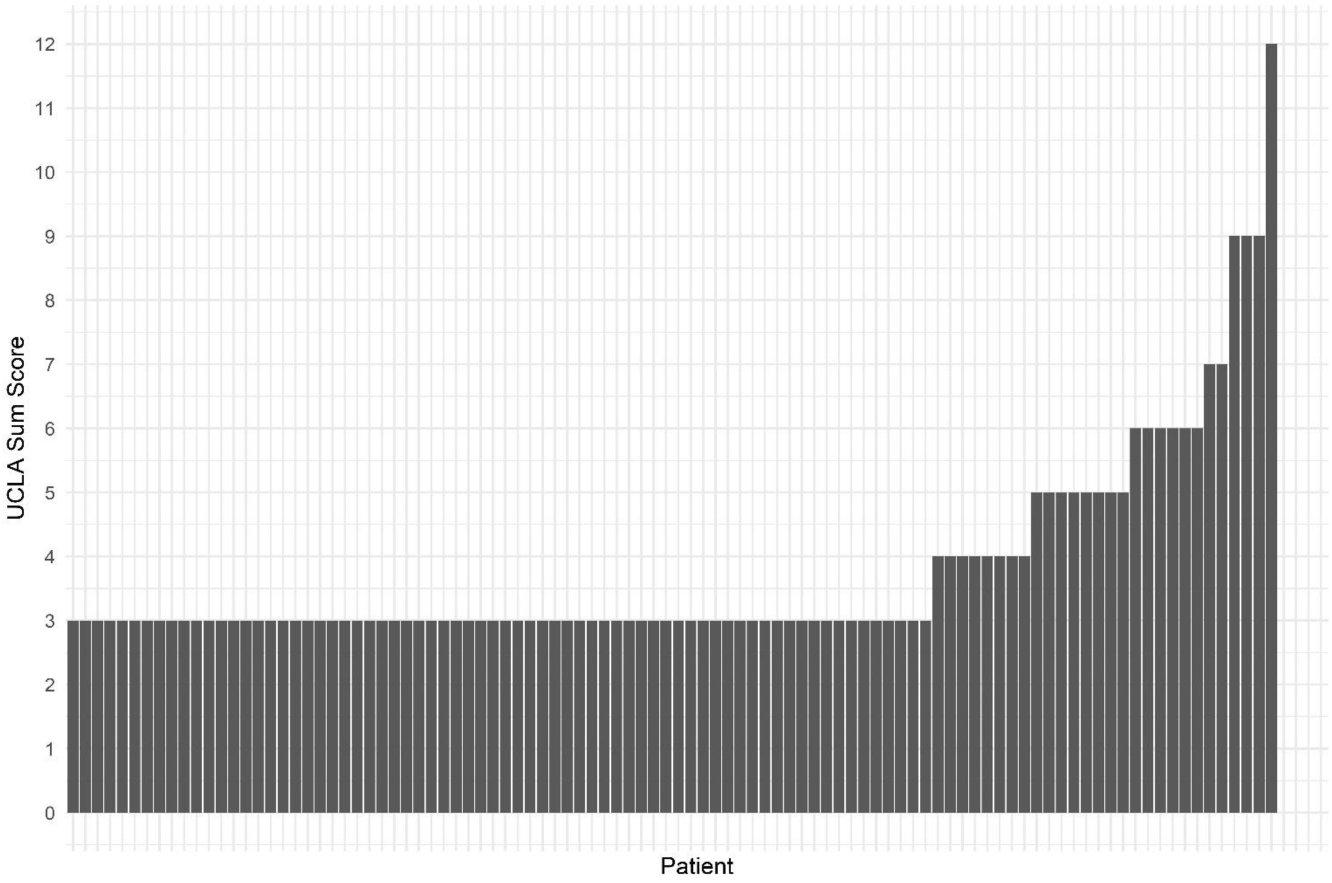

Supplement: Supplementary file 1 [file healthcare-14-00581-s001.zip › healthcare-4114658-supplementary.pdf]
